# Followee Recommendation in Microblog Using Matrix Factorization Model with Structural Regularization

**DOI:** 10.1155/2014/420841

**Published:** 2014-03-31

**Authors:** Yan Yu, Robin G. Qiu

**Affiliations:** ^1^College of Economics and Management, Nanjing University of Aeronautics and Astronautics, Nanjing 210016, China; ^2^Computer Science Department, Southeast University Chengxian College, Nanjing 210088, China; ^3^Information Science Department, Pennsylvania State University, Great Valley, Malvern, PA 16802, USA

## Abstract

Microblog that provides us a new communication and information sharing platform has been growing exponentially since it emerged just a few years ago. To microblog users, recommending followees who can serve as high quality information sources is a competitive service. To address this problem, in this paper we propose a matrix factorization model with structural regularization to improve the accuracy of followee recommendation in microblog. More specifically, we adapt the matrix factorization model in traditional item recommender systems to followee recommendation in microblog and use structural regularization to exploit structure information of social network to constrain matrix factorization model. The experimental analysis on a real-world dataset shows that our proposed model is promising.

## 1. Introduction

Microblog, such as Twitter and Google+, has become a popular Internet service. Essentially, microblog enables an easy and lightweight way of communication, which allows people to write short messages and then broadcast and share them through the participating online social networks. The short message can be anything like news, daily activities, and opinions. Microblog has noticeably changed the way of information consumption, which has surely emerged as a mainstream social network medium globally.

A large number of microblog users, together with the low cost and effort required for sharing messages, produce massive and fast growing information in microblog, giving rise to the problem of information overload. It thus becomes a great challenge for microblog users to find interesting information. One effective solution to cope with this problem is to recommend relevant and high quality information sources, that is, followees, for information seekers [1]. Surely, an effective recommending information sources service can assist information seekers to discover and connect with other users in a satisfactory manner. Promisingly, it helps increase the linkages in the social networks while significantly improving user experience in microblog. As a result, users do not leave microblog or become inactive, which is crucial to the survival and growth of microblog site in the long run [2].

Many popular microblog services such as Twitter and Tencent Weibo feature a network of followers as one of their key functions. As a result, recommending followees in microblog has become one of the hot topics and has thus attracted much research attention in recent years. Recently, due to the efficiency in dealing with traditional item recommendation, several matrix factorization methods [3–6] have been proposed for followee recommendation in microblog. Matrix factorization is a popular method in traditional item recommendation, which finds latent features for users and items by factorizing the observed user-item rating matrix and makes latent features for further predictions.

However, those methods still have several weaknesses that need to be addressed. First of all, those methods still largely follow the principle of traditional item recommendation techniques, which often rely on the user-item rating matrix [6]. Recommending followee in microblog is different from traditional item recommendation problem. The challenge of recommending followee in microblog has yet to be studied thoroughly [6]. Secondly, those methods may not capture any useful structural information of social network, which has been proven useful for recommending followee in microblog [7, 8].

Therefore, in this work, we propose a novel method using matrix factorization model bringing together structural information of social network to solve the problems mentioned above. In addition, we demonstrate the practical applicability by using a publicly available dataset and evaluate our approach.

The rest of this paper is organized as follows.  Section 2 provides some background information and reviews related research works.  Section 3 reviews popular matrix factorization model for traditional item recommendation.  Section 4 presents our proposed model. Using real-life data, Section 5 shows results of an empirical analysis.  Section 6 gives a conclusion of this paper.

## 2. Background and Related Research Works

Microblog is a web-based service that provides an easy and lightweight way of communication for users. On a microblog website, a user, that is, the follower, follows another user, known as the followee, creating an explicit following relationship. Through a formed social network which consists of users and their following relationships, a user can easily broadcast a short message to all of his/her followers and also automatically receive posts from his/her followees. Microblog has been growing in a phenomenal speed since it emerged just a few years ago. Microblog social network differs substantially from other online social networks, such as ones in Facebook or LinkedIn, where social relationships can only be established with the consent of both to be connected users. In contrast, a following relationship in microblog is asymmetric. In other words, a user can follow a followee without the followee's consent. The asymmetry of social ties in microblog has made microblog social networks be called hybrid networks [7]. They are hybrid because users create following relationships not only for communicating with friends or acquaintances but also for getting information on particular subjects [9, 10]. Kwak et al. reveal that 77.9% of users' relationships are not reciprocated in Twitter. Furthermore, 67.6% of users do not contain any reciprocation relationships, which means that the majority of users in microblog simply look for interesting information rather than keeping in touch with their friends [11]. Therefore, recommending relevant and high quality information sources to information seekers in microblog is truly a beneficial and competitive service.

**Figure 1:**
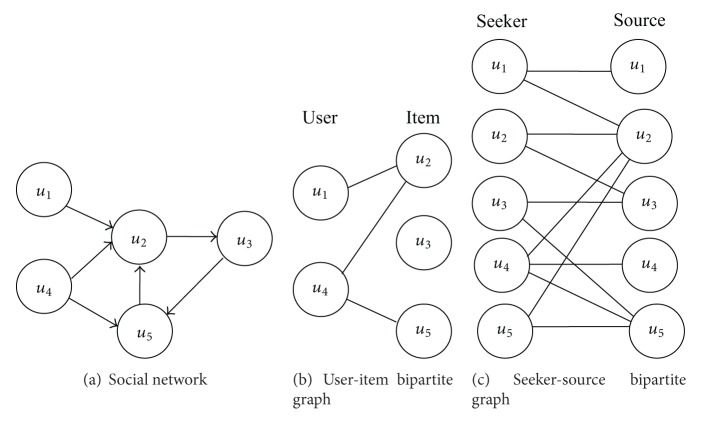
An example to illustrate seeker-source bipartite graph.

Scholars and practitioners have leveraged the rich data collected in microblog websites, such as users' posts, online social networks, actions, tags, keywords, and profiles, to help improve the effectiveness of followee recommendation in microblog [1–7, 12–15]. Among these pieces of information, the structure of social network is useful information to recommend followee in microblog [7, 8].  Figure 1(a) shows an example of social network in microblog. In this paper, we mainly focus on the followee recommendation by utilizing structure of social network of microblog.

In general, there are two main kinds of methods to make recommendations based on structure of social network. One method is the domain of* link prediction* and the other is* collaborative filtering*.* Link prediction* focuses on inferring the likelihood of the existence of a link between two nodes in a network in terms of observed link in a network. It can predict missing links or the links that may exist in the near future in a network. Technically,* link prediction* focuses on finding the most similar user to be recommended by defining the similarity among nodes based on the topology of network. For example,* Common Neighbor*,* FriendLink* [14],* PropFlow* [15], and* WTF* [2] belong to the* link prediction* approach.* Common Neighbor *measures the similarity of two nodes in the network. Intuitively, two nodes are more likely to have a link if they have many common neighbors.* FriendLink* defines a node similarity of two nodes by traversing all paths of a limited length based on the algorithmic small world hypothesis. By traversing all possible paths between a node and all other nodes in network, a node can be connected to another by many possible paths. Nodes in network can use all the pathways connecting them, proportionally to the pathway length. Thus, two nodes which are connected with many unique pathways have a high possibility to know each other, proportionally to the length of the pathways they are connected with.* PropFlow* corresponds to the probability that a restricted random walk starting at node *u* ends at a node *v* in *l* steps. The restrictions are that the walk terminates upon reaching *v* or upon revisiting any node including *u*. This produces a score that can serve as an estimation of the similarity of two nodes.* WTF* first computes the trust circle of a target user using an egocentric random walk and then constructs a bipartite graph. The bipartite graph's left side is populated with the target user's trust circle, while its right side is populated with users that the users of the trust circle follow.* WTF* runs multiple iterations of the* SALSA* algorithm to assign scores to both sides. The nodes on the right side are finally ranked in support of making recommendations.

Recently, due to the efficiency in dealing with traditional item recommendation, several* collaborative filtering* techniques [1, 3–6, 13] have been proposed for followee recommendation in microblog.* Collaborative filtering* is a technique of making automatic predictions about the interests of a user by collecting preference information from many users. There are two main categories of* collaborative filtering* techniques:* memory-based* [1, 13] and* model-based* algorithms [3–6].* Memory-based methods* mainly focus on finding the similar users for recommendation. For example, Armentano et al. propose* memory-based collaborative filtering* methods to recommend followee in microblog [1, 13]. The rationale behind the methods is that the target user is an information seeker that has already identified some interesting users acting as information sources, which are his/her current followees. Other people that also follow some of the same information sources have interests in common with the target user and might have discovered other relevant information sources in the same topics, which are in turn their followees. In contrast to the* memory-based* methods, the* model-based* approaches train a compact model by the given data and recommend followees via the trained model. For example, Chen et al. propose methods that utilize matrix factorization model to recommend followee in microblog [3–6]. Matrix factorization model is a well-known* model-based collaborative filtering* technique. Matrix factorization models have been found to be effective and efficient in traditional item recommender systems [16], which find latent features for users and items by factorizing the observed user-item rating matrix and make latent features for further predictions. Those methods using matrix factorization for followee recommendation in microblog still largely follow the principle of traditional item recommendation techniques.

Although the methods that use matrix factorization model achieve relatively good results, as mentioned in Section 1, those methods still have several weaknesses that need to be addressed. First of all, those methods for followee recommendation in microblog still largely follow the principle of traditional item recommendation techniques by viewing some users in microblog as items and the others in microblog as users, which implicitly constitutes a user-item bipartite graph.  Figure 1(b) is an example of user-item bipartite graph from Figure 1(a). User *u*_1_ and user *u*_4_ are viewed as users and users *u*_2_, *u*_3_, and *u*_5_ are viewed as items. The following relationships between users are converted into binary rating. However, recommending followee in microblog is different from traditional item recommendation problem. The first difference is that users play a dual role in microblog as they are both information sources and seekers, while users and items in traditional item recommendation play a singular role: seekers or sources. The user-item bipartite graph may lose some information. For example, in Figure 1(b) the user-item bipartite graph loses the following relationship from *u*_2_ to *u*_5_, following relationship from *u*_2_ to *u*_3_ and following relationship from *u*_3_ to *u*_5_. The second difference is that there is no explicit user-item rating in our task like traditional item recommendation. Traditional item recommendation techniques often rely on the user-item rating matrix, which explicitly represents a user's preference among items. Therefore, the challenge of recommending followee in microblog has yet to be studied thoroughly. Secondly, matrix factorization model may not capture any structural information of social network, which reflects users' preferences and is useful for followee recommendation in microblog [7, 8]. For example, in Figure 1(a), user *u*_1_ follows user *u*_2_ and *u*_2_ follows user *u*_3_; then, we will assume that *u*_1_ may be interested in *u*_3_.

We will review matrix factorization model for traditional item recommendation in Section 3, and then we will propose our method to solve the problems mentioned above in Section 4.

## 3. Matrix Factorization for Item Recommendation

Matrix factorization is a popular method in traditional item recommendation, which finds latent features for users and items by factorizing the observed user-item rating matrix and makes latent features for further predictions. Recently, due to the efficiency in dealing with traditional item recommendation, several matrix factorization methods [3–6] have been proposed for followee recommendation in microblog. Those methods still largely follow the principle of traditional item recommendation techniques. We review matrix factorization model for traditional item recommendation in this section.

Considering an *m* × *n* rating matrix *R* describing *m* users' numerical ratings on *n* items and its elements *r*_*ui*_ representing user *u*'s numerical rating on item *i*,* matrix factorization models* map users and items to a joint latent feature factor space of dimensionality *d*. Each user *u* is associated with a feature vector **p**_*u*_ ∈ ℝ^*d*^ and each item *i* is associated with a feature vector **q**_*i*_ ∈ ℝ^*d*^. The result dot product **p**_*u*_^*T*^ **q**_*i*_ represents the user *u*'s overall interest in the item *i*.* Matrix factorization models* seek to approximate **p**_*u*_ and **q**_*i*_ by minimizing the sum-of-squared-errors between *r*_*ui*_ and **p**_*u*_^*T*^ **q**_*i*_:

(1) $$\begin{array}{c} \underset{P,Q}{\min}\frac{1}{2}\sum_{u=1}^{m}\sum_{i=1}^{n}I_{ui}{\left(r_{ui}-{p_{u}}^{T}q_{i}\right)}^{2}+\frac{\lambda_{}}{2}{||P||}_{F}^{2}+\frac{\lambda_{}}{2}{||Q||}_{F}^{2}, \end{array}$$

where *P* ∈ ℝ^*d*×*m*^ is latent feature matrix of *m* users, *Q* ∈ ℝ^*d*×*n*^ is latent feature matrix of *n* items, *I*_*ui*_ is the indicator function that is equal to 1 if user *u* rated item *i* and is equal to 0 otherwise, *λ* is a nonnegative parameter of regularization term to avoid overfitting, and ||·||_*F*_^2^ denotes the Frobenius norm. A local minimum of the objective function given by (1) can be found by performing gradient descent in feature vectors **p**_*u*_ and **q**_*i*_.

## 4. Our Model

The problem we study in this paper is how to effectively and efficiently recommend Top *k* suitable followees for a user by employing the social network in microblog.

We first propose a basic matrix factorization for followee recommendation in microblog in Section 4.1. Furthermore, we use structural information of social network as structural regularization terms to constrain the matrix factorization model in Section 4.2. At last, we can get a unified model in Section 4.3.

### 4.1. Basic Matrix Factorization Model for Followee Recommendation

Microblog is essentially an information platform on which users form an explicit social network by following other users [2]. A user as a follower automatically receives the messages posted by users he/she follows, known as followees. To describe social network in microblog, we can easily construct a directed graph *G* (*U*, *E*), where *U* represents a set of users in microblog and *E* represents a set of following relationships among these users. A directed edge *u* → *v* ∈ *E* exists between users *u* and *v* if *u* follows *v*. The set of out-neighbors of user *u* is Γ_+_(*u*) = {*v* ∈ *U* | *u* → *v* ∈ *E*}, and the out-degree of *u* is |Γ_+_(*u*)|, where |·| denotes the size of the set. Similarly, Γ_−_(*u*), Γ_−_(*u*) = {*v* ∈ *U* | *v* → *u* ∈ *E*}, represents the set of in-neighbors of *u* and the in-degree of *u* is |Γ_−_(*u*)|.  Figure 1(a) shows an example of social network in microblog.

Based on the fact that users play a dual role in microblog as they are both information seekers and sources [9, 10], we characterize a user *u* in microblog with two low-dimensional feature vectors **p**_*u*_ and **q**_*u*_, which correspond to latent seeker feature vector and latent source feature vector of *u*. The inner product **p**_*u*_^*T*^ **q**_*i*_ denotes the preference of user *u* towards user *i*. Furthermore, what a user reads is often consistent with what he/she writes. We therefore assume that a user follows himself/herself. Thus, we implicitly construct a seeker-source bipartite graph from social graph in microblog.  Figure 1(c) illustrates an example of seeker-source bipartite graph from social network in Figure 1(a). For example, in Figure 1(a), user *u*_1_ follows *u*_2_; then, we can assume *u*_1_ as seeker rates *u*_1_ and *u*_2_ as sources as Figure 1(c) shows.

In this paper, unlike traditional item recommender systems, there are no explicit users' ratings on item and we need to rank the Top *k* followees for each user. So the task is actually a learning to rank task. Therefore, we formulate it as a pairwise ranking problem as Rendle et al. suggested [17]. If user *u* follows user *i*, then we assume that *u* prefers *i* over all other users which are not followed by *u*. For example, in Figure 1(a) user *u*_1_ follows user *u*_2_, but he/she does not follow *u*_4_, so we assume that *u*_1_ prefers *u*_2_ over *u*_4_, which is represented by *u*_2_>_*u*_1__ *u*_4_. For users that are both followed by a user, we cannot infer any preference. The same is true for two users that a user has not followed yet. To formalize this, we create training set *D*_*S*_ by *D*_*S*_ = {(*u*, *i*, *j*) | *u* ∈ *U*∧*i* ∈ *U*∧*j* ∈ *U*∧*u* → *i* ∈ *E*∧*u* → *j* ∉ *E*}. The semantics of (*u*, *i*, *j*) ∈ *D*_*S*_ is that user *u* is assumed to prefer *i* over *j*. The loss function specifies how close our predictions are to the actual result, and the learning methods try to minimize the loss on the training set. The loss function is

(2) min⁡P,Q L1=∑(u,i,j)∈DS−log⁡(σ(puTqi−puTqj))    +λ2||P||F2+λ2||Q||F2,

where *P* ∈ ℝ^*d*×|*U*|^ is latent seeker feature matrix of |*U*| users, *Q* ∈ ℝ^*d*×|*U*|^ is latent source feature matrix of |*U*| users, *σ* is the logistic sigmoid *σ*(*x*) = (1/(1 + *e* ^−*x*^)), *λ* represents the nonnegative parameters of regularization terms, which avoid overfitting, and ||·||_*F*_ is the Frobenius norm.

A local minimum of the objective function given by (2) can be found by performing gradient descent in feature vectors **p**_*u*_,  **q**_*i*_, and **q**_*j*_:

(3) $$\begin{array}{c} \frac{\partial L_{1}}{\partial p_{u}}=\sum_{(u,i,j)\in D_{S}}\frac{-\left(q_{i}-q_{j}\right)}{1+e^{\left({p_{u}}^{T}q_{i}-{p_{u}}^{T}q_{j}\right)}}+\lambda p_{u},\\ \frac{\partial L_{1}}{\partial q_{i}}=\sum_{(u,i,j)\in D_{S}}\frac{-p_{u}}{1+e^{\left({p_{u}}^{T}q_{i}-{p_{u}}^{T}q_{j}\right)}}+\lambda q_{i},\\ \frac{\partial L_{1}}{\partial q_{j}}=\sum_{(u,i,j)\in D_{S}}\frac{p_{u}}{1+e^{\left({p_{u}}^{T}q_{i}-{p_{u}}^{T}q_{j}\right)}}+\lambda q_{j}. \end{array}$$

Thus, we obtain a basic matrix factorization model for followee recommendation in microblog.

### 4.2. Structural Regularization

The structure of social network has proven to be useful for followee recommendation in microblog [7, 8]. In this section, we will introduce three models of structural information, that is, transitivity, similar seekers, and similar sources, as structural regularization terms to constrain the matrix factorization framework, hence generating more accurate recommendation result.  Section 4.2.1 will detail the transitivity based structural regularization.  Section 4.2.2 will introduce the similar seekers based structural regularization.  Section 4.2.3 will present similar seekers based structural regularization method.

#### 4.2.1. Transitivity Based Structural Regularization

**Figure 2:**
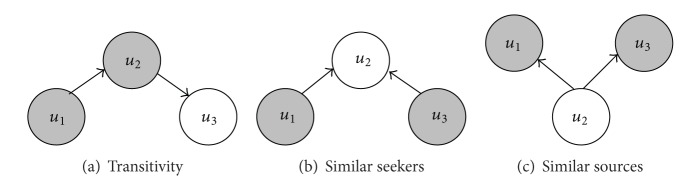
Structural regularization.

Transitivity means that in general users may be potentially influenced by their followees and tend to follow followees of their followees in microblog [7, 8]. For example, in Figure 2(a), *u*_1_ follows *u*_2_ and *u*_2_ follows *u*_3_. *u*_2_ is more likely to follow *u*_3_. Transitivity indicates that users' interests may be influenced by their followees' interests, which makes the tastes between users and their followees more similar. Based on transitivity, we could assume that user *u*'s taste **p**_*u*_ (latent seeker feature vector of *u*) should be close to the tastes of his/her followee *f* **p**_*f*_ (latent seeker feature vector of *f*). However, among all of *u*'s followees, some followees may have similar tastes with this user, while some other followees may have different tastes. We use similarity function sim(*u*, *f*) which allows the transitivity based structural regularization term to treat users' friends differently [11]. If user *u* and user *f* are very similar, then user *f* should contribute more. On the other hand, if these two users are dissimilar, then *f* should contribute less. Thus, we impose a transitivity based structural regularization term to minimize the **p**_*u*_ and **p**_*f*_ between user *u* and his/her followee *f*:

(4) $$\begin{array}{c} \frac{\alpha }{2}\sum_{u\in U}\sum_{f\in \Gamma_{+}(u)}\text{sim}\left(u,f\right){||p_{u}-p_{f}||}_{F}^{2}, \end{array}$$

where *α* is a nonnegative parameter of transitivity based structural regularization term and sim(*u*, *f*) is similarity function between user *u* and his/her follower *f* and a larger sim(*u*, *f*) value means users *u* and *f* are more similar. A small value of sim(*u*, *f*) indicates that the distance between feature vectors **p**_*u*_ and  **p**_*f*_  should be larger, while a large value tells that the distance between the latent seeker feature vectors should be smaller. In this paper, we use Jaccard Similarity Coefficient to define the similarity between two users *u* and *f* based on the followees they follow in common:

(5) $$\begin{array}{c} \text{sim}\left(u,f\right)=\frac{\left|\left(\Gamma_{+}\left(u\right)\cup \left\{u\right\}\right)\cap \left(\Gamma_{+}\left(f\right)\cup \left\{f\right\}\right)\right|}{\left|\left(\Gamma_{+}\left(u\right)\cup \left\{u\right\}\right)\cup \left(\Gamma_{+}\left(f\right)\cup \left\{f\right\}\right)\right|}. \end{array}$$

Thus, our first structural recommendation can be formulated as

(6) min⁡P,Q L2=∑(u,i,j)∈DS−log⁡(σ(puTqi−puTqj)) +α2∑u∈U∑f∈Γ+(u)sim(u,f)||pu−pf||F2 +λ2||P||F2+λ2||Q||F2.

Similar to the basic matrix factorization in Section 4.1, a local minimum of the objective function given by (6) can also be found by performing gradient descent in latent feature vectors **p**_*u*_,  **q**_*i*_, and **q**_*j*_:

(7) ∂L2∂pu=∑(u,i,j)∈DS−(qi−qj)1+e(puTqi−puTqj) +α∑f∈Γ+(u)sim(u,f)(pu−pf)+λpu,∂L2∂qi=∑(u,i,j)∈DS−pu1+e(puTqi−puTqj)+λqi,∂L2∂qj=∑(u,i,j)∈DSpu1+e(puTqi−puTqj)+λqj.

#### 4.2.2. Similar Seekers Based Structural Regularization

Similar seekers can be represented by *u*_1_ → *u*_2_ ← *u*_3_, where user *u*_1_ and user *u*_3_ all follow user *u*_2_ as Figure 2(b) shows [7, 8]. *u*_1_ and *u*_3_ each pay attention to *u*_2_, which is one kind of similarity in microblog and forms the basis for collaborative filtering algorithms, such as book and movie recommendations. Based on similar seekers, we could assume that user *u*'s taste **p**_*u*_ (latent seeker feature vector of *u*) should be close to the tastes of his/her similar seeker *n*_1_, **p**_*n*_1__ (latent seeker feature vector of *n*_1_). Thus, we impose a similar seekers based structural regularization term to minimize the **p**_*u*_ and **p**_*n*_1__ between user *u* and his/her similar seeker *n*_1_:

(8) $$\begin{array}{c} \frac{\beta }{2}\sum_{u\in U}\sum_{n_{1}\in N_{1}\left(u\right)}\text{sim}\left(u,n_{1}\right){||p_{u}-p_{n_{1}}||}_{F}^{2}, \end{array}$$

where *β* is a nonnegative parameter of similar seekers based structural regularization term, *N*_1_(*u*) is the set of Top *n* similar seekers with *u*, and sim(*u*, *n*_1_) is the same similarity we use in (5). Hence, we propose similar seekers based structural regularization term to impose constraints:

(9) min⁡P,Q L3=∑(u,i,j)∈DS−log⁡(σ(puTqi−puTqj)) +β2∑u∈U ∑n1∈N1(u)sim(u,n1)||pu−pn1||F2 +λ2||P||F2+λ2||Q||F2.

Similar to the basic matrix factorization in Section 4.1, a local minimum of the objective function given by (9) can also be found by performing gradient descent in latent feature vectors **p**_*u*_, **q**_*i*_, and **q**_*i*_:

(10) ∂L3∂pu=∑(u,i,j)∈DS−(qi−qj)1+e(puTqi−puTqj) +β∑n1∈N1(u)sim(u,n1)(pu−pn1)+λpu,∂L3∂qi=∑(u,i,j)∈DS−pu1+e(puTqi−puTqj)+λqi,∂L3∂qj=∑(u,i,j)∈DSpu1+e(puTqi−puTqj)+λqj.

#### 4.2.3. Similar Sources Based Structural Regularization

Similar sources can be represented by *u*_1_ ← *u*_2_ → *u*_3_, where user *u*_1_ and user *u*_3_ all are followed by user *u*_2_ as Figure 2(c) shows [7, 8]. If a large number of people find both *u*_1_ and *u*_3_ worthy of attention, *u*_1_ and *u*_3_ may have some shared traits driving this. Based on similar sources, we could assume that user *i*'s taste **q**_*i*_ (latent source feature vector of *i*) should be close to the tastes of his/her similar source *n*_2_, **q**_*n*_2__ (latent source feature vector of *n*_2_). Thus, we impose a similar sources based structural regularization term to minimize the **q**_*i*_ and **q**_*n*_2__ between user *u* and his/her similar source *n*_2_:

(11) $$\begin{array}{c} \frac{\gamma }{2}\sum_{i\in U}\;\sum_{n_{2}\in N_{2}(i)}\text{sim}\left(i,n_{2}\right){||q_{i}-q_{n_{2}}||}_{F}^{2}, \end{array}$$

where *γ* is a nonnegative parameter of similar sources based structural regularization term, *N*_2_(*i*) is the set of Top *n* similar sources with *i*, and sim(*i*, *n*_2_) is the same similarity we use in (5). Hence, we propose similar sources based structural regularization term to impose constraints:

(12) min⁡P,Q L4=∑(u,i,j)∈DS−log⁡(σ(puTqi−puTqj)) +γ2∑i∈U ∑n2∈N2(i)sim(i,n2)||qi−qn2||F2 +λ2||P||F2+λ2||Q||F2.

Similar to the basic matrix factorization in Section 4.1, a local minimum of the objective function given by (12) can also be found by performing gradient descent in latent feature vectors **p**_*u*_, **q**_*i*_, and **q**_*j*_:

(13) ∂L4∂pu=∑(u,i,j)∈DS−(qi−qj)1+e(puTqi−puTqj)+λpu,∂L4∂qi=∑(u,i,j)∈DS−pu1+e(puTqi−puTqj) +γ∑n2∈N2(i)sim(i,n2)(qi−qn2)+λqi,∂L4∂qj=∑(u,i,j)∈DSpu1+e(puTqi−puTqj)+λqj.

### 4.3. A Unified Model

In Section 4.1, we introduce the basic matrix factorization for recommendation in microblog. In Section 4.2, we demonstrate how to utilize structure information based on basic matrix factorization. We can then design the following integrated model to take into account all the possible information that will potentially benefit the recommendations:

(14) min⁡P,Q L5=∑(u,i,j)∈DS−log⁡(σ(puTqi−puTqj)) +α2∑u∈U ∑f∈Γ+(u)sim(u,f)||pu−pf||F2 +β2∑u∈U ∑n1∈N1(u)sim(u,n1)||pu−pn1||F2 +γ2∑i∈U ∑n2∈N2(i)sim(i,n2)||qi−qn2||F2 +λ2||P||F2+λ2||Q||F2.

Similar to the basic matrix factorization in Section 4.1, a local minimum of the objective function given by (14) can also be found by performing gradient descent in latent feature vectors **p**_*u*_,**q**_*i*_, and **q**_*j*_:

(15) ∂L5∂pu=∑(u,i,j)∈DS−(qi−qj)1+e(puTqi−puTqj) +α∑f∈Γ+(u)sim(u,f)(pu−pf) +β∑n∈N1(u)sim(u,n1)(pu−pn1) +λpu,∂L5∂qi=∑(u,i,j)∈DS−pu1+e(puTqi−puTqj) +γ∑n2∈N2(i)sim(i,n2)(qi−qn2)+λqi,∂L5∂qj=∑(u,i,j)∈DSpu1+e(puTqi−puTqj)+λqj.

The unified model is constrained by three types of structure information: transitivity, similar seekers, and similar sources based on basis matrix factorization. We use these types of structural information to help better shape the user's latent spaces and hence generate more accurate recommendation result.

## 5. Experiments

In this section, we conduct several experiments to evaluate our proposed model by utilizing a real-world dataset. More specifically, in Section 5.1, we describe the dataset and necessary experimental setup. In Section 5.2, we choose an appropriate evaluation metric.  Section 5.3 examines the performance of different models described in our paper as well as a baseline model. In Section 5.4, we explore the impact of the parameters *α*, *β*, and *γ* on the accuracy of models. Finally in Section 5.5, we report the impact of the sizes of similar seekers and similar sources on the accuracy of models.

### 5.1. Datasets and Experimental Setup

To evaluate the proposed framework, we use a real-life dataset from Tencent Weibo. Tencent Weibo is a Chinese microblog website, launched by Tencent in 2010. It has become one of leading microblog platforms in China. The dataset we use for evaluation in this paper is the dataset used in the KDD Cup 2012 Track 1 (https://www.kddcup2012.org/c/kddcup2012-track1), which is a prediction task that involves predicting whether or not a user will follow a recommended user. Track 1 in KDD Cup 2012 provides rich information across multiple domains such as user profiles, social graph, and keyword.

In this paper, we randomly sample 16,918 users and 462,485 following relationships among these sampled users. Then, we split the following relationships into two parts: training set and test set. We take the following relationships formed before November 11, 2011, as training set and the following relationships after November 11, 2011, as test set. There are totally 429,236 following relationships in training set and 33,249 following relationships in test set. Initially, we use training set for matrix factorization model training. Then, we apply the trained models on the test set to evaluate the accuracy of models.

For all the models, the dimension of latent feature is set to 50. The learning rate of matrix factorization model is set to 0.0005. The regularization parameter *λ* is set to 0.004. When a user follows *n* users, we need *n* × (16987 − *n*) pairs to evaluate for a user. In this paper, we just sample a subset of the pairs to save computation time.

### 5.2. Metrics

Researchers have used precision and average precision to evaluate the accuracy of recommendation algorithms for years. Precision measures the average percentage of the overlap between a given recommendation list and the list of followees that are actually followed. Precision can be evaluated at different points in a ranked list of recommended users. Mathematically, precision at rank *k* (*P*@*k*) is defined as the proportion of relevant users and recommended users:

(16) $$\begin{array}{c} P@k=\frac{\text{the}\;\text{number}\;\text{of}\;\text{relevant}\;\text{users}\;\text{within}\;\mathrm{rank}\;k}{k}. \end{array}$$

Average precision (AP) [1], which the KDD Cup 2012 organizers adopted, emphasizes that relevant users should be in the more forward position in the ranked list. That is, it is better to have a correct guess in the first places of the recommendation list. It is the average of precision computed at the point of each of the relevant users in the ranked list:

(17) $$\begin{array}{c} \text{AP}@k=\frac{\sum_{i=1}^{k}\left(P@i\times \mathrm{rel}\left(i\right)\right)}{\text{the}\;\text{number}\;\text{of}\;\text{relevant}\;\text{users}\;\text{with}\;k}, \end{array}$$

where *rel*⁡(*i*) is the change in the recall from *i* − 1 to *i*. MAP@*k* is the mean value of AP@*k*.

However, we think it makes more sense by considering the number and the ranking of relevant users, simultaneously. In other words, we simply replace “the number of relevant users within *k*” with “*k*” and call it AP′@*K*.

Let us use examples to illustrate the difference of applying different evaluation metrics. Assume that there are three algorithms of recommending Top 3 followees for a target user *u*_1_.  Table 1 shows the recommended followees and ones that were actually followed. Algorithms 1 and 2 have the same *P*@3, because the number of relevant users is the same. However, we intuitively think Algorithm 2 has relatively better accuracy performance than Algorithm 1, because Algorithm 2 has a correct guess in the first ranking and the second ranking. Meanwhile, Algorithm 2 and Algorithm 3 have the same AP@3. Intuitively, we know that Algorithm 3 should be better than Algorithm 2 as Algorithm 3 recommended more relevant users.

Table 1 clearly indicates that our proposed evaluation metrics can be more accurate than others. Thus, we will adopt this new evaluation metrics, AP′@*k*, which is mathematically defined as follows:

(18) $$\begin{array}{c} \text{A}{\text{P}}^{\prime }@k=\frac{\sum_{i=1}^{k}\left(P@i\times \mathrm{rel}\left(i\right)\right)}{k}. \end{array}$$

Likewise, MAP′@*k* is the mean value of AP′@*k* of all target users.

### 5.3. Models Comparisons

In this section, we compare the following different models described in this paper.

1. *User_item model*: this model uses traditional item recommendation technique by viewing some users as items and the other persons in microblog as users, which implicitly constitutes a user-item bipartite graph. Then the model uses matrix factorization modelon user_item bipartite graph to recommend followee. The details of this model are introduced in Section 3.
2. *Seeker_source model*: this is the basic matrix regularization model described in Section 4.1. We form implicitly a seeker-source bipartite graph. Then we use matrix factorization model on pairwise training set to recommend.
3. *Seeker_source_tran model*: this is the transitivity based structural regularization model using user and his/her followee information to constrain latent seeker feature vectors, which is described in Section 4.2.1. The transitivity based structural regularization parameter *α* is set to 1.
4. *Seeker_source_n*_1_ *model*: this is the similar seekers based structural regularization modelusing similar seekers' information to constrain latent seeker feature vectors between similar seekers, which is described in Section 4.2.2. Thesimilar seekers based structural regularization parameter *β* is set to 1, and the size of similar seekers is equal to 50.
5. * Seeker_source_n*_2_ *model*: this is the similar source based structural regularization model using similar source information to constrain latent source feature vectors between similar sources, which is described in Section 4.2.3. The similar sources based structural regularization parameter *γ* is set to 1, and the size of similar sources is equal to 50.
6. *Seeker_source_uni model*: this is the unified model discussed in Section 4.3, which uses followees, similar seekers, and similar sources information to constrain latent feature vectors. We use the setting *α* = *β* = *γ* = 1, and the sizes of similar seekers and similar sources are set to 50.

The results of comparisons are stated in Table 2.

From the results, we first observe that* Seeker_source model* outperforms* User_item model*.* User_item model* uses traditional item recommendation technique by viewing some persons as items and others as users in microblog, while* Seeker_source model* describes a user in microblog with two feature vectors which correspond to the information seeker and information source in microblog. As we describe before,* User_item model* may lose some following relationships. Furthermore, as there are no explicit ratings,* Seeker_source model* uses pairwise ranking as objective function. The result is consistent with our intuition.

Secondly, we find that three structural information based models are better than basic* Seeker_source model*. More specifically, among* Seeker_source model* and* Seeker_source_tran model*, we notice that the latter generates better result than the former, which demonstrates that there exists preference transitivity between users and their followees. The preference transitivity information can be utilized to constrain the latent feature vectors, such that* Seeker_source_tran model* improves the accuracy of recommendation compared with* Seeker_source model*. Among* Seeker_source model* and* Seeker_source_n*_1_ *model*, we observe that the latter is better than the former.* Seeker_source_n*_1_ *model* utilizes the implicit similar seekers' information to constrain the latent seeker feature vectors, such that* Seeker_source_n*_1_ *model* can improve the accuracy of recommendation compared with* Seeker_source model*. Among* Seeker_source model* and* Seeker_source_n*_2_ model, the latter is better than the former.* Seeker_source_n*_2_ model utilizes the implicit similar sources information to constrain the latent seeker feature vectors, such that* Seeker_source_n*_2_ *model* can improve the accuracy of recommendation compared with* Seeker_source model*. So, we can find that transitivity, similar seekers, and similar sources are all useful structural information which can be utilized to improve the accuracy of recommendation.

Lastly, an integrated model,* Seeker_source_uni,* demonstrates the best performance by incorporating all the useful transitivity, similar seekers, and similar sources.

In summary, combining basic matrix factorization model with the help of structure regularization, the proposed model outperforms baseline model and its variants. In the next two sections, we investigate more details about the impact of structural regularization on the proposed model.

### 5.4. Impact of Parameters *α*, *β*, and *γ*

**Figure 3:**
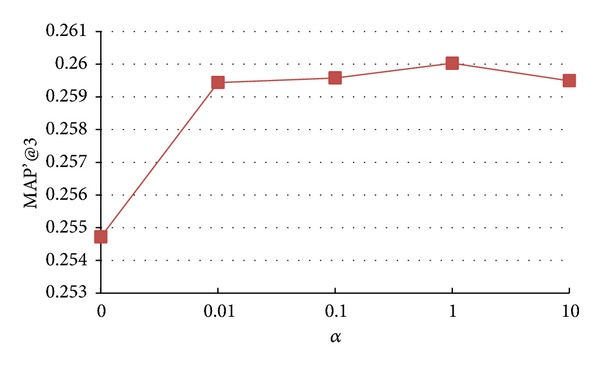
Impact of parameter *α* on accuracy of* Seeker_source_tran model*.

**Figure 4:**
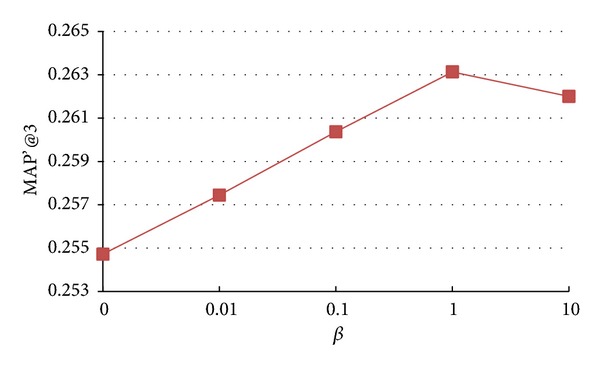
Impact of parameter *β* on accuracy of* Seeker_source_n*_*1*_ *model*.

**Figure 5:**
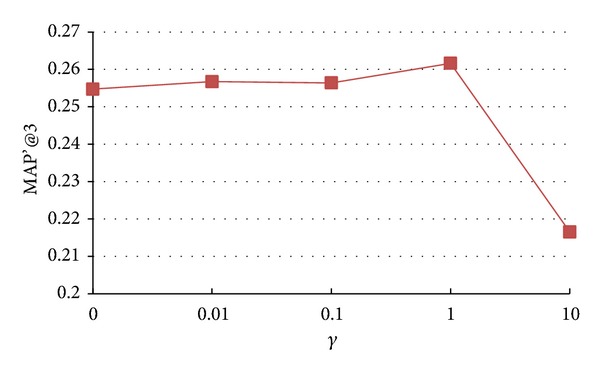
Impact of parameter *γ* on accuracy of* Seeker_source_n*_*2*_ *model*.

In this paper, the parameters *α*, *β*, and *γ* are introduced to control the extent of three structural regularization terms. In the extreme case, if *α*, *β*, and *γ* are set to very small values, the structural regularization contributes a little to model learning process. On the other hand, if we set large values to *α*, *β*, and *γ*, the structural information will dominate the model learning process. In this section, we analyze how the changes of *α*, *β*, and *γ* affect the final recommendation accuracy. We vary the value of *α*, *β*, and *γ* as {0, 0.01, 0.1, 1, 10, 100} and the results of* Seeker_source_tran model*,* Seeker_source_n*_*1*_ *model, *and* Seeker_source_n*_*2*_ *model* are shown in Figures 3, 4, and 5, respectively.

From Figures 3, 4, and 5, we observe that, in general, with the increase of *α*, *β*, and *γ*, the performance shows similar trends: first increasing, reaching peak values, and then decreasing. More specifically, as *α*, *β*, and *γ* increase, the accuracy of* Seeker_source_tran*,* Seeker_sourc_n*_1,_  and* Seeker_source_n*_2_  models increases at first, but when *α*, *β*, and *γ* reach 1, the accuracy of three models decreases with further increase of the value of *α*, *β*, and *γ*. The results demonstrate that we should control the extent of structural regularization properly. Incorporating properly the structural information can improve the recommendation accuracy, while the inappropriate extent of integration of structural information may cause the accuracy of recommendation to degrade.

### 5.5. Impact of Sizes of Similar Seekers and Similar Sources

**Figure 6:**
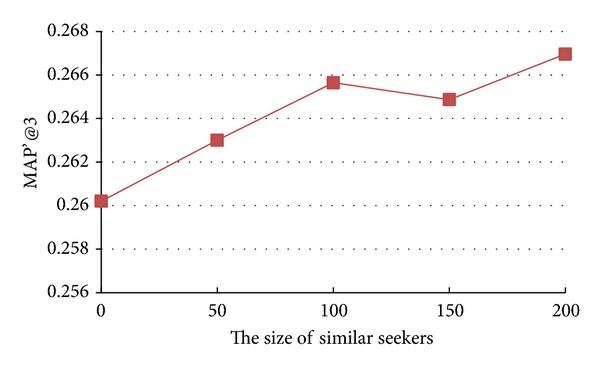
Impact of size of similar seekers on accuracy of* Seeker_source_n*_*1*_ *model*.

**Figure 7:**
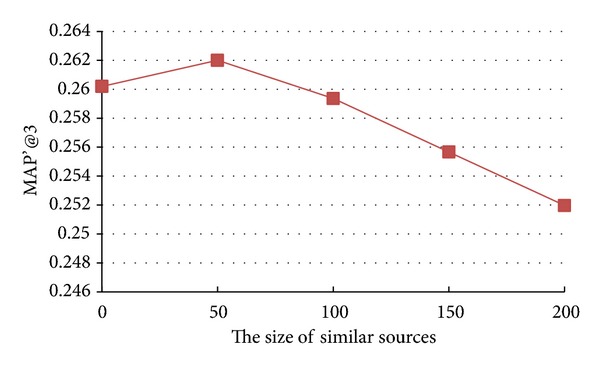
Impact of size of similar sources on accuracy of* Seeker_source*_*n*_2_ *model. *

The models we study in this paper also involve the calculation of the Top *n* similar seekers or similar sources. In this section, we analyze how the changes of sizes of similar seekers and similar sources can affect the recommendation accuracy. We vary the value of sizes of similar seekers and similar sources as {0, 50, 100, 150, 200} and the results of* Seeker_source_n*_*1*_ *model* and* Seeker_source_n*_*2*_ *model* are shown in Figures 6 and 7.

From Figures 6 and 7, we observe two different patterns. From Figure 6, we notice that, with the increase of size of similar seekers, the accuracy of* Seeker_source_n*_1_ *model* tends to increase. On the contrary, with the increase of size of similar sources, the accuracy of* Seeker_source_n*_2_ *model* first increases, reaching peak values, and then decreases as in Figure 7.

These two different patterns may be that most users tend to follow celebrities in Tencent Weibo. Compared with a large number of common users, the number of celebrities is very small. For example, in our experimental dataset, there are 2,857 celebrities among all 16,918 users. It is easier to find 200 similar seekers in 16,918 users than to find 200 similar sources in 2,857 celebrities. The results demonstrate that truly similar seekers and similar sources can improve the accuracy of recommendation, but integrating not similar seekers or sources may degrade the accuracy of recommendation.

## 6. Conclusion and Future Work

Microblog that provides us a new communication and information sharing platform has been growing exponentially since it emerged just a few years ago. To microblog users, recommending followees who can serve as high quality information sources is a competitive service, which not only helps increase the linkages in the social networks but also enriches user's experience in microblogging. To address this problem, in this paper we propose a new model to improve the accuracy of followee recommendation in microblog. More specifically, we propose a model that adapts the matrix factorization model in traditional item recommender systems for followee recommendation in microblog and integrates the structural information of social network. The experimental analysis on a real-world dataset shows that our proposed model is promising. In light of our future study, we are planning to include some other information from microblog to further improve the accuracy of followee recommendation.

## Figures and Tables

**Table 1:** Differences of applying different evaluation metrics.

Algorithm	Target user	Recommended user	Accepted user	*P*@3	AP@3	AP′@3
Algorithm 1	*u*_1_	*u*_2_ *u*_4_ *u*_3_	*u*_2_ *u*_3_	2/3 = 0.667	(1/1 + 2/3)/2 = 0.833	(1/1 + 2/3)/3 = 0.556

Algorithm 2	*u*_1_	*u*_2_ *u*_3_ *u*_4_	*u*_2_ *u*_3_	2/3 = 0.667	(1/1 + 2/2)/2 = 1.000	(1/1 + 2/2)/3 = 0.667

Algorithm 3	*u*_1_	*u*_2_ *u*_3_ *u*_5_	*u*_2_ *u*_3_ *u*_5_	3/3 = 1.000	(1/1 + 2/2 + 3/3)/3 = 1.000	(1/1 + 2/2 + 3/3)/3 = 1.000

**Table 2:** Accuracy comparisons of different models.

	MAP′@1	MAP′@3	MAP′@5	MAP′@10
*User*_*item *	0.422	0.252	0.184	0.118
*Seeker*_*source *	0.424	0.255	0.186	0.119
*Seeker*_*source*_*tran *	0.427	0.260	0.190	0.121
*Seeker*_*source*_*n*_1_	0.432	0.263	0.193	0.122
*Seeker*_*source*_*n*_2_	0.439	0.262	0.192	0.121
*Seeker*_*source*_*uni *	0.442	0.266	0.195	0.123
